# Isolated Pancreatic Tuberculosis Mimicking Pancreatic Cancer: A Diagnostic Challenge

**DOI:** 10.1155/2018/7871503

**Published:** 2018-04-12

**Authors:** Abhishek Bhurwal, Muhammad Masoodul Haq, Sunil Sapru, Matthew Tortora, Dhanasekaran Ramasamy

**Affiliations:** ^1^Department of Internal Medicine, Saint Barnabas Medical Center, RWJBarnabas Health, West Orange, NJ, USA; ^2^Department of Pathology, Saint Barnabas Medical Center, RWJBarnabas Health, West Orange, NJ, USA; ^3^Department of Internal Medicine, Division of Gastroenterology, Saint Barnabas Medical Center, RWJBarnabas Health, West Orange, NJ, USA

## Abstract

Isolated pancreatic tuberculosis is an exceedingly rare condition, even in areas of the world where the disease is highly prevalent. Abdominal tuberculosis is a common form of extrapulmonary tuberculosis but involvement of the pancreas is very rare. We report a case of isolated pancreatic tuberculosis presenting as a pancreatic mass in a patient with persistent abdominal pain and jaundice. Clinically and radiologically, the mass mimicked a malignant pancreatic tumor with a vastly different prognostic implication and therapeutic approach. Endoscopic ultrasound with fine-needle aspiration (EUS-FNA) can provide valuable diagnostic information in this scenario. After the tissue showed evidence of acid-fast bacilli and the cultures showed growth of* Mycobacterium tuberculosis*, antituberculosis therapy was initiated. Conservative management is usually successful in alleviating symptoms and leading to a cure. The excellent response to ATT makes it imperative that these patients are diagnosed early and managed appropriately to avoid unnecessary surgery and associated morbidity.

## 1. Background

Isolated pancreatic tuberculosis is an exceedingly rare condition, even in areas of the world where the disease is highly prevalent [1]. The diagnosis is often a challenge as the symptoms and radiological appearance often mimic pancreatic cancer. Endoscopic ultrasound with fine-needle aspiration (EUS-FNA) can provide valuable diagnostic information [2]. The excellent response to antitubercular therapy makes it imperative to diagnose pancreatic tuberculosis early and avoid unnecessary interventions. We report one such case of isolated pancreatic tuberculosis presenting as a pancreatic mass where cytological diagnosis prevented untoward surgical resection.

## 2. Case Report

A previously healthy 38-year-old gentleman began to experience episodes of intermittent abdominal pain five days prior to presentation. The abdominal pain was predominantly in the epigastrium, nonradiating, and occasionally associated with nausea. He did not have any episodes of vomiting or diarrhea. Over the course of next five days, he noticed yellowing of his eyes and urine which prompted him to visit the emergency room. Patient denied any history of fever, night sweats, weight loss, or decreased appetite. Family history was unremarkable. He migrated to the United States at the age of 33 from the Philippines. He had received BCG vaccination during childhood but had no previous history of treatment for tuberculosis. He denied any sick contacts or travel within the last few years. He worked as a pharmacist. He denied smoking and alcohol or intravenous drug use.

The patient appeared well nourished with icteric sclera. Physical examination revealed a temperature of 38°C, blood pressure 110/55 mmHg, heart rate 85 beats per minute, respiratory rate 18 breaths per minute, and oxygen saturation 95% on room air. Cardiopulmonary examination was normal. Abdominal examination revealed tenderness in the epigastric region with no guarding or rigidity. However, no palpable mass could be appreciated.

Routine laboratory investigations showed normochromic normocytic anemia with Hb 12.3 gm/dl. Total bilirubin was 2.9 mg/dl while aspartate aminotransferase 167 U/L, alanine aminotransferase 443 U/L, and alkaline phosphatase were 424 U/L, respectively. Serum lipase and amylase were not elevated.

**Figure 1:**
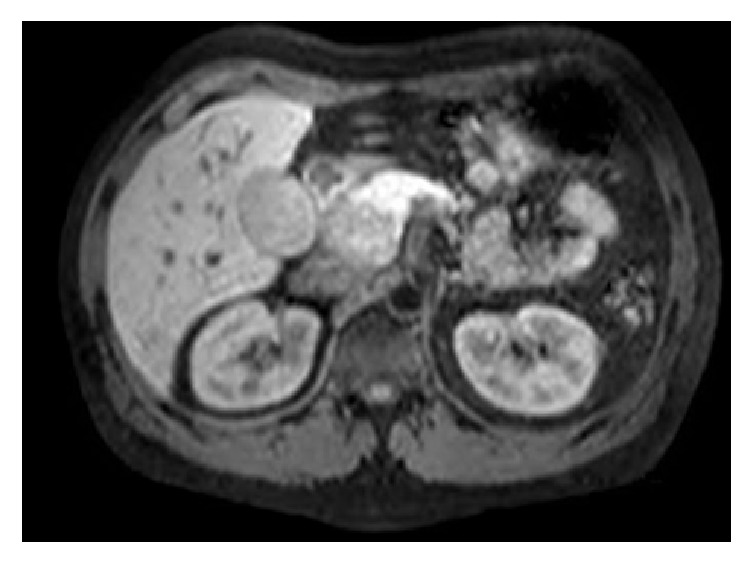
Initial MRCP showing pancreatic head mass with nonenhancing areas in the center.

The chest radiograph at the time of admission was normal. Subsequently, he underwent an abdominal computed tomography (CT) scan which revealed a 3.8 × 2.7 cm solid mass posterolateral to the pancreatic head, suggestive of peripancreatic lymphadenopathy. Magnetic resonance imaging (MRCP) showed a 4.1 × 2.6 cm mass in the pancreatic head encasing the portal vein with multiple central areas of nonenhancement (Figure 1). There was a 1.5 cm segment of narrowing in the mid common bile duct with mild dilation of the intrahepatic ducts. The pancreatic duct was not dilated.

**Figure 2:**
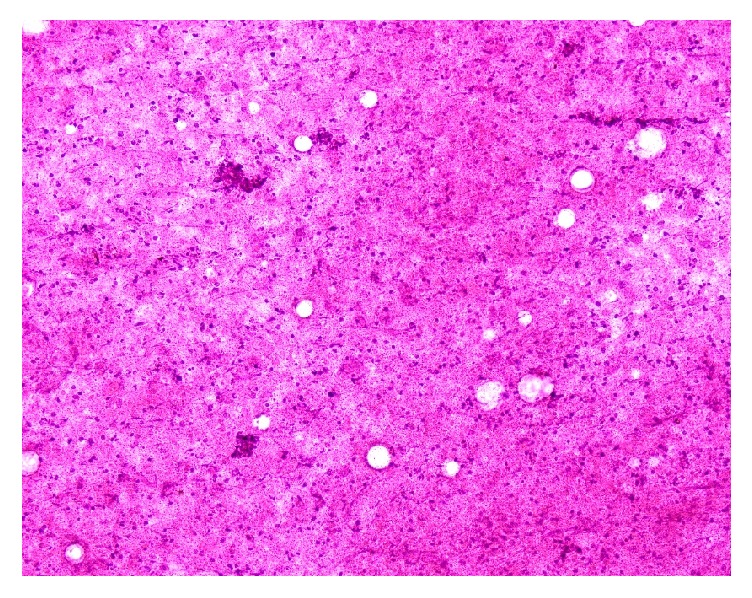
Necrotic material on the needle aspiration smear (H&E stain).

The differential diagnosis included malignant pancreatic tumor, autoimmune pancreatitis, neuroendocrine tumor, and gastrointestinal stromal tumor. Endoscopic ultrasound (EUS) with fine-needle aspiration (FNA) was performed which showed a heterogeneous hyperechoic pancreatic lesion which on histopathology revealed areas of coagulation necrosis, lymphocytes, and epithelioid histiocytes suggestive of granulomatous inflammation (Figure 2).

## 3. Discussion

Granulomatous inflammation in the pancreas is an unusual occurrence and has rarely been described in the literature [6]. It can be seen in mycobacterial infections, fungal infections, sarcoidosis, inflammatory bowel disorder, Wegner's disease, malignancy, and foreign body retention after procedures [6]. In our patient, fungal infection was unlikely with negative HIV and no immunosuppression, along with no evidence of systemic involvement. He did not have any prior ERCP or other procedures to suspect foreign body retention in the pancreas. Absence of sinus disease, bland urinalysis, and no kidney involvement ruled out Wegner's disease. Crohn's disease was unlikely due to absence of bowel involvement. Our patient did not have any febrile episodes, night sweats, weight loss, or exposure to sick contacts and there was no pulmonary involvement to suggest mycobacterial infection. His only risk factor was that he was born in Philippines.

**Figure 3:**
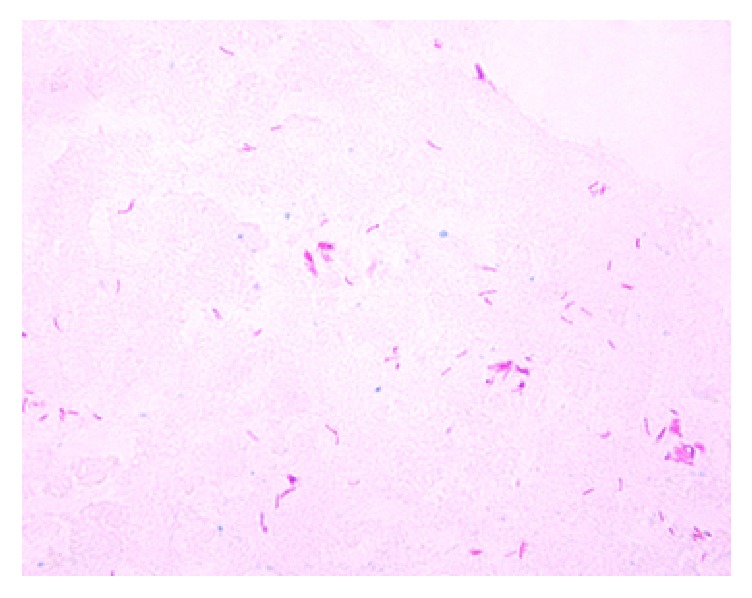
Scattered acid-fast bacilli on the cell block preparation (acid-fast stain).

**Figure 4:**
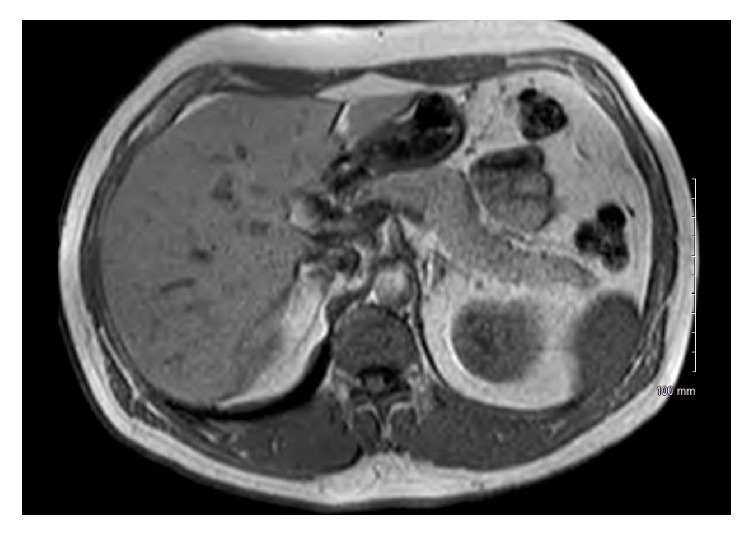
Follow-up imaging at 5 months showing resolution with no discrete pancreatic head mass.

Even though our initial suspicion was low, the tissue showed evidence of acid-fast bacilli and the cultures showed growth of* Mycobacterium tuberculosis* (Figure 3). After the diagnosis of tuberculosis was confirmed by AFB stain and cultures, he was started on antitubercular therapy. He was treated with isoniazid, rifampin, pyrazinamide, and ethambutol for 4 months followed by isoniazid and rifampin for 10 months. He tolerated the regimen well. Repeat laboratory investigations such as total bilirubin level and liver chemistries (AST, ALT, and ALP) were also normal. Repeat MRI performed at 5 months of treatment did not reveal any discrete pancreatic lesion (Figure 4).

Tuberculosis is a common occurrence in certain endemic zones of Asia and Africa [7]. Even though, tuberculosis primarily affects the lungs, isolated abdominal tuberculosis is not rare with incidence ranging from 0.59% to 12% [1, 8]. Pancreatic tuberculosis was first described by Auerbach in 1944 [9]. Since then, most of the medical literature on this rare disease is limited to case reports or small case series.

The possible mechanisms of isolated pancreatic tuberculosis are as follows [10]:

- Hematogenous dissemination to the pancreas from an occult lesion in the lungs or abdomen
- Direct spread from contiguous lymph nodes
- Reactivation of a dormant bacilli in an old tubercular lesion in an immunosuppressive state.

Pancreatic tuberculosis most commonly presents with nonspecific symptoms such as abdominal pain, jaundice, vomiting, anorexia, or weight loss. It usually involves the head and uncinate process of the pancreas. Most of the cases have been described in young people, especially females, often with a past history of TB or coming from endemic zone of active tuberculosis [11].  Table 1 shows recently published case reports of isolated pancreatic tuberculosis in the United States. Our case is only the fourth case of isolated pancreatic tuberculosis in the Western hemisphere over the last 5 years [3–5].

The diagnosis of pancreatic tuberculosis is a challenge because of the rarity of the disease and nonspecific signs and symptoms of the disease. Ultrasound and CECT are often used for initial evaluation. Focal hypoechoic lesions or cystic lesions are usually seen on ultrasonography [12]. CT may reveal hypodense lesions mostly in the head of the pancreas [13]. There might be diffuse enlargement of the pancreas or presence of enlarged peripancreatic lymph nodes [13]. There are no specific features on imaging and even the evidence of vascular invasion does not differentiate malignancy from pancreatic tuberculosis [10].

The suspicion of malignancy leads to an unwarranted resection as many cases are diagnosed after surgical resection [14]. Therefore, high index of suspicion is warranted to diagnose the condition early to prevent unnecessary surgical intervention and associated morbidity. EUS-FNA, CT/US guided biopsy or surgical biopsy provides valuable histopathological diagnosis [2]. The presence of caseating granulomatous inflammation and acid-fast bacilli is suggestive of tuberculosis. Cultures for mycobacteria take up to 6 weeks to grow and are used to confirm the diagnosis. Once the diagnosis is confirmed, medical management (antitubercular therapy (ATT)) is the mainstay of the therapy. ATT consists of multidrug chemotherapy and is usually recommended for 6 to 12 months [15]. The disease responds well to the treatment.

In summary, isolated pancreatic tuberculosis is an uncommon presentation of tuberculosis. The clinical presentation and the radiological finding of a pancreatic mass may be suggestive of a malignant pancreatic tumor with a vastly different prognostic implication and therapeutic approach. The diagnosis of isolated pancreatic TB requires a high index of suspicion. It should be considered in patients presenting with pancreatic mass especially in immunocompromised patients. EUS-FNA is an excellent investigation to detect pancreatic tuberculosis promptly and prevents unnecessary surgical procedures. Patients respond well to conservative management and antitubercular therapy.

## Figures and Tables

**Table 1:** It is showing cases of isolated pancreatic tuberculosis in the Western World along with the case characteristics and management strategies and outcomes over the last 5 years.

Study, year	Patient characteristics	Therapy and outcomes
Patel et al., 2013 [3]	(i) 41-year-old female (ii) CT, 5.1 cm × 2.8 cm multiloculated solid lesion in the head of the pancreas (iii) EUS, 4.3 cm × 2.5 cm hypoechoic, homogenous mass lesion with a cystic component within the pancreatic head	RIPE for 2 months and then IR for 7 months

Salahuddin and Saif, 2014 [4]	(i) 59-year-old male (ii) MRCP, dilated common bile duct, dilated pancreatic duct, and enlarged lymph nodes in the porta hepatis, peripancreatic and perigastric regions (iii) EUS, ahypoechoic mass measuring 3 by 4 cm was seen in the pancreatic head	Antitubercular therapy (RIPE) for 18 months

Waintraub et al., 2016 [5]	(i) 31-year-old male (ii) MRI, periportal lymphadenopathy indenting pancreatic head and uncinate process and compressing CBD (iii) EUS, irregular, hypoechoic mass with poorly defined borders in the pancreatic head. Anechoic lesion in the pancreatic tail, suggestive of a cyst or pseudocyst.	Antitubercular therapy

## References

[1] Pramesh C. S., Heroor A. A., Gupta S. G. (2003). Pancreatic tuberculosis: An elusive diagnosis. *HPB*.

[2] Riaz A. A., Singh A., Robshaw P., Isla A. M. (2002). Tuberculosis of the pancreas diagnosed with needle aspiration. *Infectious Diseases*.

[3] Patel D., Loren D., Kowalski T., Siddiqui A. A. (2013). Pancreatic tuberculosis mimicking malignancy diagnosed with endoscopic ultrasound-guided fine needle aspiration. *Endoscopic Ultrasound*.

[4] Salahuddin A., Saif M. W. (2014). Pancreatic tuberculosis or autoimmune pancreatitis. *Case Reports in Medicine*.

[5] Waintraub D. J., D’Souza L. S., Madrigal E., Harshan M., Ascunce G. I. (2016). A Rare Case of Isolated Pancreatic Tuberculosis. *ACG Case Reports Journal*.

[6] Stürmer J., Becker V. (1987). Granulomatous pancreatitis - granulomas in chronic pancreatitis. *Virchows Archiv A Pathological Anatomy and Histopathology*.

[7] Floyd K., Pantoja A. (2008). Financial resources required for tuberculosis control to achieve global targets set for 2015. *Bulletin of the World Health Organization*.

[8] Rong Y., Lou W., Jin D. (2008). Pancreatic tuberculosis with splenic tuberculosis mimicking advanced pancreatic cancer with splenic metastasizes: a case report. *Cases Journal*.

[9] Auerbach O. (1944). Acute Generalized Miliary Tuberculosis. *American Journal of Pathology*.

[10] Liu Q., He Z., Bie P. (2003). Solitary pancreatic tuberculous abscess mimicking prancreatic cystadenocarcinoma: A case report. *BMC Gastroenterology*.

[11] Xia F., Poon T.-P., Wang S.-G., Bie P., Huang X.-Q., Dong J.-H. (2003). Tuberculosis of pancreas and peripancreatic lymph nodes in immunocompetent patients: experience from China. *World Journal of Gastroenterology*.

[12] Morris D. L., Wilkinson L. S., Mokhtar N. A. (1993). Case report: Emphysematous tuberculous pancreatitis diagnosis by ultrasound and computed tomography. *Clinical Radiology*.

[13] Pombo F., Díaz Candamio M. J., Rodriguez E., Pombo S. (1998). Pancreatic tuberculosis: CT findings. *Abdominal Imaging*.

[14] Saluja S. S., Ray S., Pal S. (2007). Hepatobiliary and pancreatic tuberculosis: a two decade experience. *BMC Surgery*.

[15] Chaudhary P., Bhadana U., Arora M. P. (2015). Pancreatic Tuberculosis. *Indian Journal of Surgery*.

